# Prevalence of Dementia in China in 2015: A Nationwide Community-Based Study

**DOI:** 10.3389/fpubh.2021.733314

**Published:** 2021-11-02

**Authors:** Shige Qi, Peng Yin, Han Zhang, Qingjun Zhang, Yize Xiao, Ying Deng, Zhong Dong, Yan Shi, Jun Meng, Dantao Peng, Zhihui Wang

**Affiliations:** ^1^National Center for Chronic and Noncommunicable Disease Control and Prevention, Chinese Center for Disease Control and Prevention, Beijing, China; ^2^Hubei Provincial Center for Disease Control and Prevention, Wuhan, China; ^3^Yunnan Center for Disease Control and Prevention, Kunming, China; ^4^Sichuan Center for Disease Control and Prevention, Chengdu, China; ^5^Beijing Center for Disease Control and Prevention, Beijing, China; ^6^Shanghai Municipal Center for Disease Control and Prevention, Shanghai, China; ^7^Guangxi Center for Disease Control and Prevention, Nanning, China; ^8^China-Japan Friendship Hospital, Beijing, China

**Keywords:** dementia, prevalence, risk factors, Alzheimer's disease, cross-sectional study

## Abstract

**Objective:** This study aims to estimate the prevalence of dementia and Alzheimer's disease (AD) and associated risk factors among the general Chinese population.

**Methods:** We carried out a nationwide study including 24,117 participants aged 60 years and older in China using a multistage clustered sampling. Dementia and AD were diagnosed according to the fourth edition of the Diagnostic and Statistical Manual of Mental Disorders and the criteria issued by the National Institute of Neurological and Communicative Disorders and Stroke–Alzheimer's Disease and Related Disorders Association. Face-to-face interviews were administered by the trained interviewers to obtain information on demographics, lifestyle factors, and previous diseases.

**Results:** The overall weighted prevalence of dementia was 4.22% (95%CI 2.27–6.17%) for people aged 60 years and older, was higher in women than in men and increased with age. Daily tea drinking and daily exercises were the protective factors for both dementia and AD. Engaging in social and intellectual activities was significantly associated with a lower risk of dementia and AD.

**Conclusions:** A large number of population with dementia posed a significant challenge to China where the population is rapidly aging. The increase of public awareness, building more care facilities, and training dementia specialists and professional caregivers are all urgently needed and should be the future priorities of dementia care in China.

## Introduction

China is aging much faster than other low- and middle-income countries. WHO estimated that the proportion of the population aged 60 years and over will increase from 12.4% in 2010 to 28% in 2040 (1). Accompanying the aging of the society is an increase in age-associated diseases, with dementia playing an important role. The World Alzheimer Report 2015 estimated that there were over 9.9 million new cases of dementia each year worldwide, implying one new case every 3.2 s (2). The Global Burden of Disease Study 2016 reported that the number of patients with dementia in China accounted for ~25% of the entire population with dementia worldwide (3). A few studies have reported the prevalence of dementia in China, and the estimates remain inconsistent, ranging from 5.0 to 7.7% for people aged 60 years and older and from 2.0 to 13.0% for people aged 65 years and older (4). Most of the previous studies were carried out in single cities or a few localities with small sample sizes, and the significant variations might be mainly due to the different diagnostic criteria, instruments used to assess dementia, the time of the study, and a different sampling scheme, and the characteristics of the study participants. A multicity cross-sectional study reported that the prevalence was 5.60% (3.50–7.60%) for people aged 65 years and above in 2013, based on 5,326 study participants (5). A recent meta-analysis summarized 96 studies and reported an overall prevalence of dementia of 5.30% (4.30–6.30%) for Chinese population aged 60 years and above (6), which was lower than the estimate for China (6.19%) and southeast Asia (7.64%) by the World Alzheimer Report 2015 (2).

In an aging society such as China, an understanding of the current prevalence of dementia and its risk factors is important for policymakers to prioritize patient care and allocate limited health resources. In this article, we reported the prevalence of dementia and Alzheimer's disease (AD) and associated risk factors among the general population aged 60 years and older, based on a large nationwide study carried out in China in 2015.

## Methods

### Study Population

**Figure 1:**
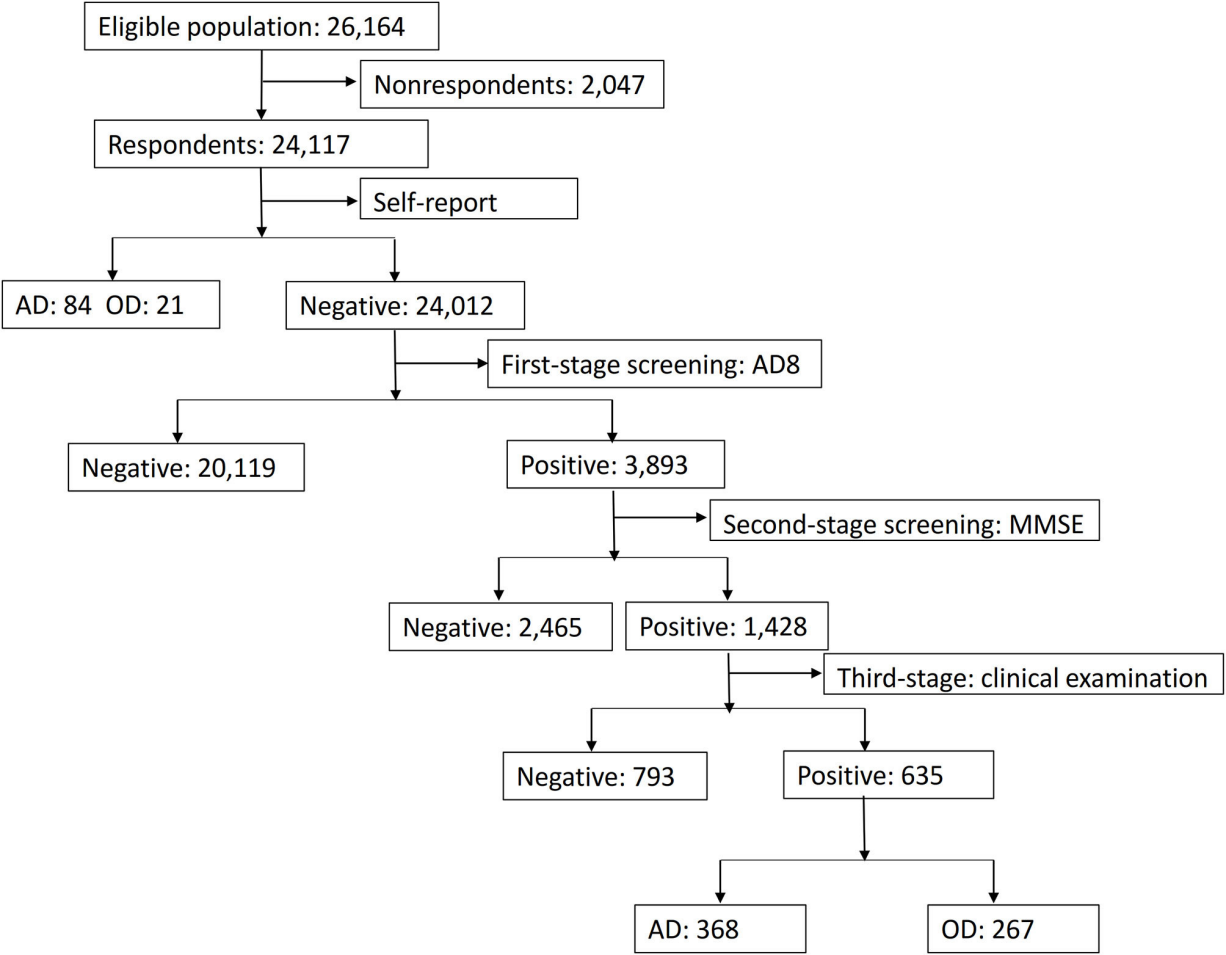
Study flow chart. AD, Alzheimer's disease; OD, other types of dementia; AD8, Ascertain Dementia 8; MMSE, Mini-Mental State Examination.

The Prevention and Intervention on Neurodegenerative Disease for Elderly in China (PINDEC) study was initiated in 2015 aiming to understand the epidemiology of neurodegenerative diseases and associated risk factors among the population aged 60 years and above in China. We used multistage clustered sampling to select the study population. According to the geographic location, population size, and level of economic development, we first selected Beijing, Shanghai, Hubei, Sichuan, Guangxi, and Yunnan as study provinces, representing 20.7% of the total Chinese population and 22.2% of the population aged 60 years and above in 2015 (Appendix 1). Within each province, one urban district and one rural county were randomly selected as the study sites (counties or districts). Within each site, one subdistrict in urban areas or one township in rural areas was selected with probability proportional to its size. Within each subdistrict or township, four to eight neighborhood communities or administrative villages were selected with probability proportional to their size. Within each neighborhood community or administrative village, 100–200 households with people aged 60 years and above were randomly selected as the study households. In the final stage, study participants were selected based on the inclusion and exclusion criteria. The inclusion criteria are as follows: (1) aged 60 years and above (2) have registered Hukou, and (3) lived in the household for more than 1 year. The Hukou system is a family registration program that serves as a domestic passport in China, regulating population distribution and rural-to-urban migration. All individuals register their Hukou in Public Security Department as a legal document, and the Hukou statistics are normally used as a reliable and stable source to reflect the local demography as it is not biased by a rapidly growing number of the migrant population. Subjects who refused to participate, had a life-threatening illness, were living in hospitals, or were institutionalized were excluded. Figure 1 shows the flow chart of this study. A total of 26,164 people were selected, and 24,117 participated in the survey. The overall response rate was 92.2%. The main reasons for refusal included participants who were too busy, temporarily visited families living in other cities, or they think the survey is not important to themselves.

### Data Collection

Data collection was conducted either in examination centers at local health stations in the residential area of participants or in the household of participants by the trained staff according to a standard protocol, from June to December 2015. A comprehensive questionnaire, including information on demographic characteristics, medical history, and lifestyle factors, was administered by the trained interviewers. Bodyweight, height, and waist circumference were measured according to a standard protocol, and body mass index (BMI) was calculated as the weight in kilograms divided by the height in square meter. Based on the Chinese criteria of the China Obesity Working Group (7), normal weight was defined as 18.5 ≤ BMI < 24.0, low weight was defined as BMI < 18.5, overweight was defined as 24.0 ≤ BMI < 28.0, and obesity was defined as BMI ≥ 28.0. Blood pressure was measured at the non-dominant arm three times consecutively with a 1-min interval between the measurements in a seating position after 5 min of rest using an automated device. Blood samples were collected in all participants after overnight fasting of at least 10 h. Fasting blood glucose (FBG) and serum total cholesterol (TC), low-density lipoprotein-cholesterol (LDL-C), high-density lipoprotein cholesterol (HDL-C), and triglycerides (TG) were measured. Diabetes was defined as (1) a self-reported previous diagnosis by healthcare professionals, and (2) the FBG level of 126 mg/dl (7.0 mmol/L) or higher (8). The 2016 Chinese Guideline for the Management of Dyslipidemia in Adults (Chinese guideline) (9) was used to classify the serum TC, LDL-C, HDL-C, and TG levels. High TC was defined as TC ≥ 6.22 mmol/L. High LDL-C was defined as LDL-C ≥ 4.14 mmol/L. Low HDL-C was defined as LDL-C < 1.04 mmol/L and high TG was defined as TG ≥ 2.26 mmol/L. The presence of other diseases was a self-reported previous diagnosis by healthcare professionals.

To ensure the validity and reliability of the data, a stringent quality control protocol was implemented throughout the data collection process. All investigators, interviewers, and laboratory staff underwent intensive training sessions with written and practical exams after the training. Only certified staff were approved to participate in this study and to carry out data collection in the field sites. All questionnaires were administered using a portable PAD with automatic skip pattern and logic checking. All interviews were recorded, and 5% of the total recordings were randomly selected to check consistency and data coding quality.

### Dementia and AD Assessment

When the study participant was selected in the final sampling stage, all participants were asked whether they have been previously diagnosed with dementia by health professionals. Medical records were obtained for those with an affirmative answer, and a self-reported diagnosis was made if the diagnosis was made with clinical examinations in hospitals confirmed by the interviewers. The rest of the participants underwent a three-stage approach for dementia assessment. They were first screened with a Chinese version of the Ascertain Dementia 8 (AD8), which has been proven to be a useful and simple screening tool with good validity in the Chinese population (93.9% sensitivity and 76.0% specificity) (10). Participants with an AD8 score of ≥2 were then assessed with Mini-Mental State Examination (MMSE), and cognitive impairment was defined as MMSE ≤ 17 for illiterate, ≤ 20 for primary school, and ≤ 24 for junior high school and above (11, 12). In the final stage, all participants with a cognitive impairment underwent a thorough clinical examination by neurologists. Dementia was diagnosed based on the fourth edition of the Diagnostic and Statistical Manual of Mental Disorders (13). The diagnosis of AD was made based on the criteria issued by the National Institute of Neurological and Communicative Disorders and Stroke–Alzheimer's Disease and Related Disorders Association (14). A regional expert committee comprised of neurologists at each of the six provinces was available to review all the diagnoses, and a national working committee gathered together to discuss difficult cases until a consensus was reached.

### Statistical Analysis

There were very few missing data in the questionnaires, and they were imputed with median or mean values for the same gender and age group in the dataset. Demographic characteristics of study participants and associated factors were described in means (95% CIs) for continuous variables and percentages (95% CIs) for categorical variables in the overall population and in the subgroups of sex, location (urban/rural), age, and educational level. The prevalence of dementia and AD was estimated separately for the overall population and for subgroups. All estimates were weighted to represent the overall Chinese population aged 60 years or older. We calculated sampling weighting, non-response weighting, and post-stratification weighting to derive a final weighing for each study participant. The detailed weighting procedure and the results were shown in Appendix 2. To compare with the prevalence from previous studies, we also estimated the prevalence of dementia for people aged 65 years and over. Multivariable logistic regression was used to examine the association of demographic, lifestyle, and metabolic factors with the odds of dementia and AD. We presented the results for both the crude and fully adjusted models. All *P p-*values were two-tailed, and the *p*-value of < 0.05 was considered statistically significant. All statistical analyses were conducted using the SAS system, version 9.4 (SAS Institute, Inc., Cary, NC, USA).

## Results

The characteristics of study participants are presented in Table 1. Among the 24,117 people aged 60 years and above included in the analysis, 44.5% were men, 53.7% were in urban areas, and 21.1% were widowed. We found no statistically significant difference between men and women with respect to living in urban/rural areas and living alone or with family. The mean age was 70.5 years (SD 7.0), and the median age was 69.0 years. The current smoking rate was 40.3% for men and 2.1% for women. The mean BMI was 23.5 kg/m^2^, and 80.1% had regular exercise. About 70.7% socialized with neighbors on daily basis, and 92.9% almost never used the internet. The prevalence of diabetes was 19.0%, and the prevalence of high TC, high LDL-C, low HDL-C, and high TG was 10.2, 7.0, 12.1, and 15.8%, respectively.

**Table 1:** General characteristics of the study population in 2015, *n* (%).

	**Total**	**Men**	**Women**	***P-*value**
Overall	24,117 (100.0)	10,722 (100.0)	13,395 (100.0)	
**Age groups, years**				0.008
60–64	5,346 (22.2)	2,232 (20.8)	3,114 (23.2)	
65–69	7,033 (29.2)	3,142 (29.3)	3,891 (29.0)	
70–74	5,076 (21.0)	2,353 (21.9)	2,723 (20.3)	
75–79	3,639 (15.1)	1,647 (15.4)	1,992 (14.9)	
≥80	3,023 (12.5)	1,348 (12.6)	1,675 (12.5)	
**Location**				0.816
Urban	12,950 (53.7)	5,553 (51.8)	7,397 (55.2)	
Rural	11,167 (46.3)	5,169 (48.2)	5,998 (44.8)	
**Marital status**				<0.001
Married	18,613 (77.2)	9,194 (85.7)	9,419 (70.3)	
Single/divorced/separated	418 (1.7)	303 (2.8)	115 (0.9)	
Widowed	5,086 (21.1)	1,225 (11.4)	3,861 (28.8)	
**Educational level**				<0.001
Illiterate	9,376 (38.9)	2,676 (25.0)	6,700 (50.0)	
Primary school	7,652 (31.7)	4,077 (38.0)	3,575 (26.7)	
Junior high school	4,546 (18.8)	2,504 (23.4)	2,042 (15.2)	
Junior high school and above	2,543 (10.5)	1,465 (13.7)	1,078 (8.0)	
**Occupation**				0.002
Farmer	14,049 (58.2)	5,580 (52.1)	8,469 (63.2)	
Worker	5,760 (23.9)	2,932 (27.3)	2,828 (21.1)	
Non-manual worker^*^	4,308 (17.9)	2,210 (20.6)	2,098 (15.7)	
Current smoker	4,601 (19.1)	4,325 (40.3)	276 (2.1)	<0.001
Regular exercise	19,323 (80.1)	8,762 (81.7)	10,561 (78.8)	0.005
**Daily tea drinking**				<0.001
Green tea	4,935 (20.5)	3,500 (32.6)	1,435 (10.7)	
Black tea	1,488 (6.2)	951 (8.9)	537 (4.0)	
Alcohol drinking	5,344 (22.2)	4345 (40.5)	999 (7.5)	<0.001
BMI (kg/m^2^)_	23.5 ± 0.02	23.2 ± 0.03	23.7 ± 0.03	<0.001^#^
**Living status**				0.056
Alone	2,824 (11.7)	1,057 (9.9)	1,767 (13.2)	
With family	21,293 (88.3)	9,665 (90.1)	11,628 (86.8)	
**Socializing with neighbors**				0.002
Almost never	3,567 (14.8)	1,802 (16.8)	1,765 (13.2)	
Occasional	3,506 (14.5)	1,666 (15.5)	1,840 (13.7)	
Daily	17,044 (70.7)	7,254 (67.7)	9,790 (73.1)	
**Reading newspapers**				<0.001
Almost never	17,329 (71.9)	6,716 (62.6)	10,613 (79.2)	
Occasional	3,493 (14.5)	1,993 (18.6)	1,500 (11.2)	
Daily	3,295 (13.7)	2,013 (18.8)	1,282 (9.6)	
**Use of internet**				0.001
Almost never	22,393 (92.9)	9,792 (91.3)	12,601 (94.1)	
Occasional	783 (3.2)	405 (3.8)	378 (2.8)	
Daily	941 (3.9)	525 (4.9)	416 (3.1)	
Diabetes	19.0	17.9	19.9	0.050
**Dyslipidemia**				
High TC	2,457 (10.2)	781 (7.3)	1,676 (12.5)	0.001
High LDL-C	1,697 (7.0)	569 (5.3)	1,128 (8.4)	<0.001
Low HDL-C	2,923 (12.1)	1,521 (14.2)	1,402 (10.5)	0.014
High TG	3,802 (15.8)	1,404 (13.1)	2,398 (17.9)	<0.001

* *Non-manual worker includes teacher, researcher, doctors, office workers, and other occupations apart from farmer and worker*.

# *ANOVA was used to compare the difference of body mass index (BMI) between men and women*.

The overall weighted prevalence of dementia was 4.22% (95%CI 2.27–6.17%), 2.04% (95%CI 1.54–2.55%) in men and 6.32% (95%CI 2.77–9.86%) in women, 2.90% (95%CI 1.61–4.19%) in urban areas, and 5.26% (95%CI 2.91–7.60%) in rural areas. The prevalence increased with age from 1.95% (95%CI 0.97–2.92%) in 60–64 years to 9.46% (95%CI 7.11–11.81%) in the age group 80 years and above. The prevalence of dementia was the highest in the illiterate group, decreased with more years of education but increased again in people with the highest educational level (more than 8 years). The weighted prevalence of AD was 2.32% (95%CI 1.60–3.05%), and the pattern in different subgroups was similar to dementia (Table 2). The weighted prevalence of dementia and AD for people aged 65 years and over was 5.34%, 95%CI 3.08–7.61% and 2.94%, 95%CI 2.10–3.78%, respectively (Appendix 3). There were substantial geographic variations in the weighted prevalence of dementia with the highest in Sichuan province (6.25%, 95%CI 5.50–7.00%) and the lowest in Guangxi province (1.05%, 95%CI 0.73–1.37%) (Appendix 4).

**Table 2:** The estimated prevalence of dementia and Alzheimer's disease (AD) in the Chinese population.

	**Dementia**		**AD**	
	**No**.	**Weighted prevalence % (95%CI)**	**No**.	**Weighted prevalence % (95%CI)**
Overall	740	4.22 (2.27–6.17)	452	2.32 (1.60–3.05)
**Sex**				
Men	220	2.04 (1.54–2.55)	147	1.41 (1.03–1.78)
Women	520	6.32 (2.77–9.86)	305	3.20 (2.00–4.40)
**Age groups, years**				
60–64	73	1.95 (0.97–2.92)	46	1.07 (0.71–1.43)
65–69	129	2.76 (1.21–4.31)	65	1.14 (0.71–1.57)
70–74	130	3.80 (1.88–5.72)	70	1.93 (1.03–2.84)
75–79	188	8.31 (3.85–12.77)	110	3.93 (2.78–5.08)
≥80	220	9.46 (7.11–11.81)	161	6.91 (4.60–9.22)
**Location**				
Urban	356	2.90 (1.61–4.19)	214	1.78 (0.96–2.59)
Rural	384	5.26 (2.91–7.60)	238	2.75 (2.04–3.45)
**Marital status**				
Married	485	3.36 (1.85–4.88)	291	1.80 (1.27–2.33)
Single/divorced/separated	16	3.19 (2.12–4.26)	12	2.54 (1.32–3.75)
Widowed	239	7.37 (4.01–10.73)	149	4.16 (2.58–5.74)
**Education, years**				
Illiterate	454	7.26 (3.02–11.5)	268	3.92 (2.35–5.50)
Primary school	157	2.19 (1.36–3.03)	102	1.22 (0.75–1.68)
Junior high school	80	1.96 (1.18–2.75)	47	1.02 (0.42–1.62)
Junior high school and above	49	2.32 (1.73–2.91)	35	1.67 (1.17–2.16)

**Figure 2:**
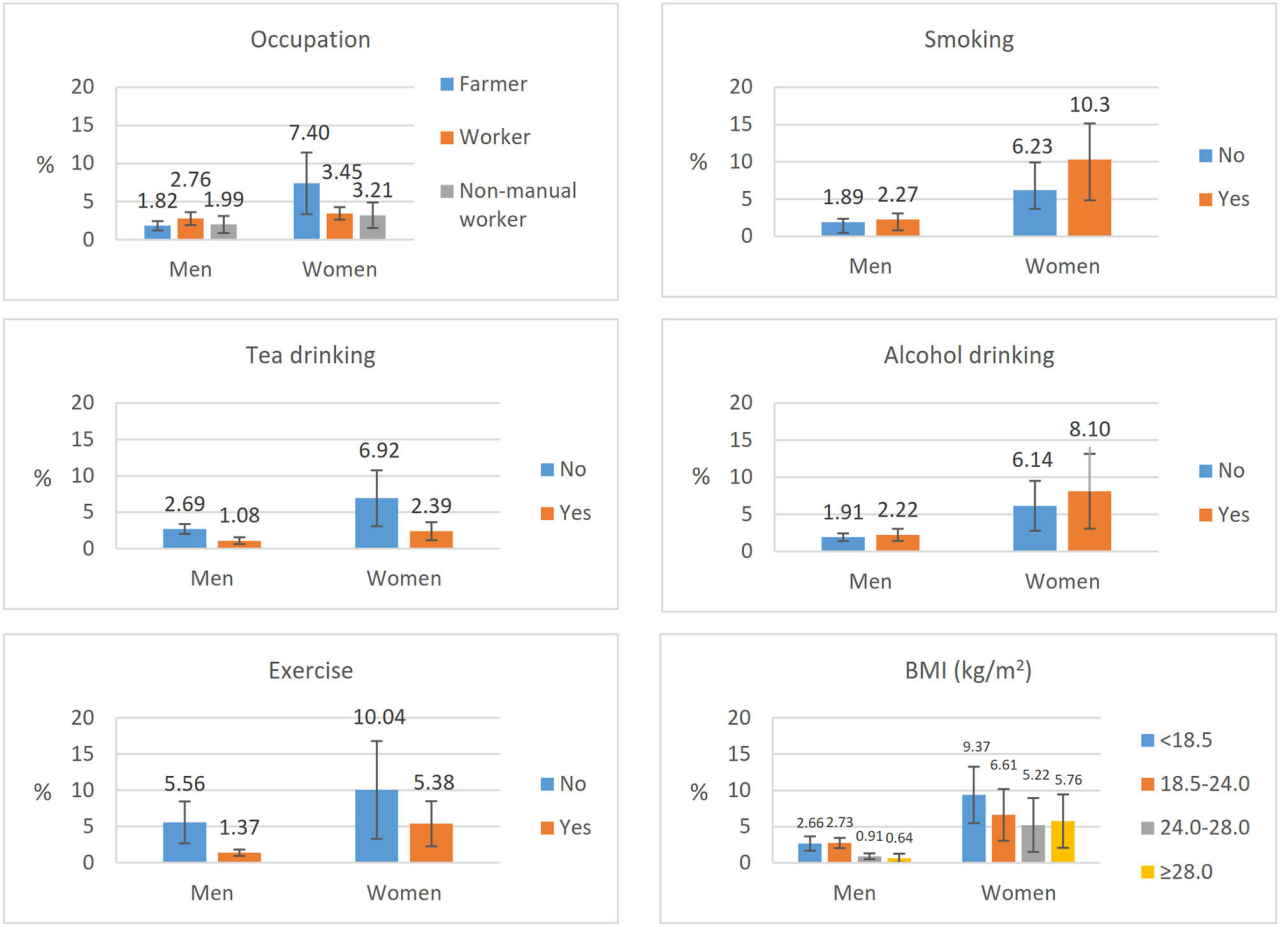
The estimated prevalence of dementia stratified by behavioral and metabolic risk factors.

Figure 2 shows the estimated prevalence rate of dementia by different behavioral and metabolic risk factors. The prevalence of dementia was lower in daily tea drinkers than those who did not drink tea on daily basis and was lower in people with daily exercises than those who did not have regular exercise for both men and women. The prevalence of dementia was the highest in low BMI and the lowest in those with the obesity group for women, and the highest in normal BMI, and the lowest in the overweight group for men. The differences were not statistically significant in terms of smoking status and alcohol drinking. We tested the interaction for different risk factors with gender, and the results showed that there were interactions for occupation (*p* = 0.006), BMI (*p* = 0.011), and regular exercises (*p* < 0.0001) with gender, but not for the others.

Table 3 shows the odds ratios (OR) of different risk factors for dementia and AD. Age was a strong risk factor for dementia and AD. The educational level at primary school (6 years) was associated with a lower prevalence rate compared with illiterate people. However, when the educational level is increasing to junior high school or above, the association was no longer significant. Regular tea drinking, daily exercises, daily socializing with neighbors, reading newspapers or books on a daily basis, and daily use of the internet were the protective factors for both dementia and AD. Engaging in intellectual activities was significantly associated with a lower risk of dementia and AD. Smoking was associated with a higher prevalence of AD (adjusted OR 1.51, 95%CI 1.12–2.04) but not with dementia. Alcohol drinking, the presence of diabetes, and the presence of stroke were associated with a higher prevalence of dementia but not with AD. The presence of chronic obstructive pulmonary disease (COPD) and arthritis was both associated with a higher prevalence of dementia and AD.

**Table 3:** Logistic regression analysis of potential risk factors for dementia and AD (odds ratios (OR), 95%CI).

	**Dementia**		**AD**	
	**Model 1**	**Model 2**	**Model 1**	**Model 2**
**Sex**				
Men (ref)	1.00	1.00	1.00	1.00
Women	**3.06 (1.70–5.51)**	**2.79 (2.05–3.80)**	**2.15 (1.51–3.07)**	**1.83 (1.24–2.69)**
**Age groups, years**				
60–64 (ref)	1.00	1.00	1.00	1.00
65–69	**1.43 (1.13–1.82)**	**1.40 (1.14–1.72)**	1.07 (0.74–1.54)	1.04 (0.73–1.49)
70–74	**1.99 (1.63–2.43)**	**1.86 (1.55–2.21)**	**1.82 (1.27–2.61)**	**1.67 (1.18–2.36)**
75–79	**4.57 (3.66–5.71)**	**3.53 (2.96–4.21)**	**3.79 (3.32–4.32)**	**2.85 (2.22–3.66)**
≥80	**5.27 (3.76–7.38)**	**3.17 (2.21–4.55)**	**6.87 (5.40–8.73)**	**4.42 (3.51–5.55)**
**Education, years**				
Illiterate (ref)	1.00	1.00	1.00	1.00
Primary school	**0.35 (0.22–0.56)**	**0.53 (0.42–0.67)**	**0.39 (0.23–0.66)**	**0.52 (0.34–0.79)**
Junior high school	**0.32 (0.17–0.63)**	0.82 (0.62–1.08)	**0.34 (0.15–0.78)**	0.76 (0.43–1.33)
Junior high school and above	**0.33 (0.15–0.72)**	1.05 (0.66–1.66)	**0.46 (0.27–0.78)**	1.43 (0.84–2.43)
**Residing status**				
Alone (ref)	1.00	1.00	1.00	1.00
Living with family	0.81 (0.67–1.00)	0.96 (0. 82–1.13)	0.72 (0.54–1.00)	**0.78 (0.64–0.96)**
**Smoking**				
Never (ref)	1.00	1.00	1.00	1.00
Ever	0.67 (0.33–1.36)	1.24 (0.93–1.66)	0.95 (0.62–1.46)	**1.51 (1.12–2.04)**
**Alcohol drinking**				
No (ref)	1.00	1.00	1.00	1.00
Yes	0.83 (0.68–1.02)	**1.51 (1.25–1.83)**	0.81 (0.60–1.09)	1.19 (0.91–1.56)
**Daily tea drinking**				
No (ref)	1.00	1.00	1.00	1.00
Green tea	**0.28 (0.21–0.37)**	**0.54 (0.43–0.68)**	**0.27 (0.19–0.37)**	**0.48 (0.36–0.62)**
Black tea	**0.20 (0.08–0.50)**	**0.33 (0.15–0.72)**	**0.25 (0.12–0.50)**	**0.37 (0.19–0.74**
**Regular exercise**				
No (ref)	1.00	1.00	1.00	1.00
Yes	**0.47 (0.33–0.68)**	**0.63 (0.42–0.95)**	**0.52 (0.36–0.77)**	**0.72 (0.49–0.97)**
**Socializing with neighbors**				
Almost never (ref)	1.00	1.00	1.00	1.00
Occasional	0.74 (0.40–1.40)	0.73 (0.45–1.19)	0.65 (0.39–1.07)	0.69 (0.46–1.04)
Daily	**0.41 (0.24–0.70)**	**0.44 (0.27–0.73)**	**0.47 (0.26–0.82)**	**0.54 (0.33–0.88)**
**Reading newspapers**				
Almost never (ref)	1.00	1.00	1.00	1.00
Occasional	**0.22 (0.06–0.78)**	0.44 (0.16–1.20)	0.36 (0.13–1.02)	0.62 (0.27–1.44)
Daily	**0.14 (0.05–0.38)**	**0.31 (0.13–0.73)**	**0.11 (0.04–0.30)**	**0.19 (0.07–0.48)**
**Use internet**				
Almost never (ref)	1.00	1.00	1.00	1.00
Occasional	**0.15 (0.05–0.46)**	**0.40 (0.18–0.91)**	**0.12 (0.02–0.98)**	0.29 (0.03–2.95)
Daily	**0.14 (0.05–0.41)**	**0.33 (0.17–0.64)**	**0.21 (0.08–0.55)**	**0.42 (0.20–0.90)**
**Diabetes**				
No (ref)	1.00	1.00	1.00	1.00
Yes	**1.43 (1.15–1.79)**	**1.22 (1.01–1.48)**	1.24 (0.95–1.60)	1.00 (0.81–1.23)
**High TC**				
No (ref)	1.00	1.00	1.00	1.00
Yes	1.19 (0.70–2.01)	0.71 (0.46–1.10)	1.67 (0.92–3.01)	1.02 (0.56–1.84)
**High LDL-C**				
No (ref)	1.00	1.00	1.00	1.00
Yes	**1.82 (1.10–3.00)**	1.56 (0.98–2.49)	**2.39 (1.39–4.11)**	1.72 (0.96–3.06)
**Low HDL-C**				
No (ref)	1.00	1.00	1.00	1.00
Yes	1.23 (0.77–1.95)	1.17 (0.91–1.49)	1.17 (0.82–1.66)	0.99 (0.74–1.33)
**High TG**				
No (ref)	1.00	1.00	1.00	1.00
Yes	0.92 (0.60–1.40)	0.81 (0.54–1.21)	0.95 (0.65–1.40)	0.84 (0.50–1.41)
**CHD**				
No (ref)	1.00	1.00	1.00	1.00
Yes	**1.89 (1.19–3.00)**	1.24 (0.94–1.65)	**2.07 (1.45–2.95)**	1.31 (0.99–1.72)
**Stroke**				
No (ref)	1.00	1.00	1.00	1.00
Yes	**2.91 (1.86–4.57)**	**2.21 (1.62–3.01)**	**2.23 (1.27–3.92)**	1.35 (0.78–2.32)
**Cataract**				
No (ref)	1.00	1.00	1.00	1.00
Yes	1.52 (0.94–2.43)	1.07 (0.75–1.52)	**1.86 (1.14–3.04)**	1.27 (0.82–1.98)
**COPD**				
No (ref)	1.00	1.00	1.00	1.00
Yes	**2.06 (1.38–3.08)**	**1.94 (1.48–2.54)**	**2.19 (1.54–3.12)**	**1.84 (1.46–2.32)**
**Arthritis**				
No (ref)	1.00	1.00	1.00	1.00
Yes	**1.83 (1.41–2.36)**	**1.44 (1.23–1.69)**	**2.01 (1.54–2.61)**	**1.60 (1.21–2.11)**

*Model 1: Unadjusted crude analysis*.

*Model 2: Adjusted for age, sex, marital status, region, educational level, occupation, smoking status, alcohol drinking, tea drinking, BMI, exercise, residing status, socializing with neighbors, reading newspapers, use of the internet, and the presence of some diseases*.

*Bold values: P-value < 0.05*.

Appendix 5 shows the estimated prevalence of dementia using the different forms of dyslipidemia. The prevalence of dementia was higher in men with high TC, high LDL-C, and low HDL-C than those without these diseases. The dementia rate was higher in women with high TG than those without a high TG level. However, when controlling the confounding factors in the logistic regression analysis, the association between dementia and the different forms of dyslipidemia was no longer significant.

## Discussion

In this large-scale community-based survey recently carried out in China, we found that the prevalence of dementia and AD was 4.22% (95%CI 2.27–6.17%) and 2.32% (95%CI 1.60–3.05%) in individuals aged 60 years and above in 2015. The prevalence increased with age and was higher in women than in men. Regular tea drinking, daily exercises, and frequent participation in social and intellectual activities were significantly associated with a lower prevalence of dementia and AD.

Systematic review articles all reported the pooled rates of dementia/AD among older adults aged 60 or above, and our estimates (4.22%) fall within the range of 3.0–5.3% reported in these studies (6, 15, 16). In comparison with the latest study from Jia et al. (4), our estimates on the prevalence of dementia for individuals aged 60 years and above were in the lower range of previous estimates. This might be explained by our study sample. We randomly selected the study participants from communities across the urban and rural areas in China and represented the general Chinese population. In comparison with other studies, our estimate on Chinese population aged over 60 years was higher than a meta-analysis in 2007 and 2012 (15, 16) and our estimates on Chinese population aged over 65 years (5.35%) were in keeping with a five-city study (17) (5.14%) in 2008–2009 and the multicity study (5) in 2012 (5.6%). When we further compared the prevalence rate among different 5-year age groups, we found our estimates for the 65–69, 70–74, and 75–79 age groups were all comparable with the estimates from Jia et al. (17) for both urban and rural areas. However, our estimates for the age group 80 years and above were remarkably lower, resulting in our overall estimates in the lower range of the previous estimates. This can be mainly explained that the elderly participants in our study were relatively healthy because our sample was selected from the general population and excluded those in hospitals or institutionalized.

In consistent with previous studies, we observed a significantly higher prevalence in rural areas than in urban areas, in women than in men, for both dementia and AD. The prevalence of dementia in women was particularly higher than the prevalence of dementia in men, compared with a recent large-scale cross-sectional study (18). The magnitude of the differences might be due to different study populations and settings, and further studies are needed to fully address the discrepancy. The prevalence of dementia and AD among the widowed participants was more than 2-fold of the non-widowed participants, which is also in line with previous reports from both developed countries and China (19–21). Educational level has been identified as an important factor associated with dementia, and illiterate individuals had the highest prevalence rate of dementia and different types of dementia, as indicated in many previous studies (22, 23). Jia et al. reported that the prevalence of AD decreased with more years of education in urban areas in China, while in rural areas, the prevalence was the lowest for those with 7–9 years of education and slightly higher in those with more than 9 years of education (17).

Accumulated evidence from prospective cohort studies proved an increased risk of dementia in smokers. Our study found that smoking was associated with a higher prevalence of AD but not for dementia. Another multicity study in China found no association between tobacco smoking and the prevalence of AD and vascular dementia in urban and rural Chinese populations (17). The reason remains unclear, and further studies with biomarker assessment for smoking are needed to illustrate the true association between smoking and dementia in the Chinese population. Previous studies indicated that the association between alcohol consumption and the risk of dementia is dependent on the amount of alcohol (24, 25). Although the information on the quantity of alcohol was not obtained in the current study, an analysis on frequency showed that daily alcohol drinking was significantly associated with a higher risk of dementia. For tea-drinking, our result showed that both green tea and black tea were the protective factors for dementia and AD. A few studies have reported the hypothesis that green tea intake might reduce the risk for dementia, AD (26). However, whether black tea intake was associated with dementia and AD needs further research.

Previous studies found that rheumatoid arthritis (RA) increased the risk of dementia, and also reported that a decreased risk of new-onset dementia was seen in patients with RA and was greater among older men, which may be due to the use of the antirheumatic drug (27, 28). In our study, the measurement for arthritis only used self-report, and we did not collect the information about arthritis type and medical history. Potential reasons can be further explored in future studies. Consistent with many studies, a higher prevalence of dementia and AD was seen in patients with COPD (29–31).

The most recent World Alzheimer Report 2019 indicated that 80% of the general public are concerned about developing dementia at some point, and one in four people think that there is nothing we can do to prevent dementia, and almost 62% of the healthcare providers worldwide think that dementia is part of normal aging (32). According to a recent national WeChat-based (a popular instant messaging app) survey on public knowledge about dementia in China, the overall correct rate of total dementia knowledge was 63% and only half of the participants could identify risk factors accurately (33). Their findings indicated that Chinese people have a low level of knowledge about dementia, especially those aged more than 60 years, with low education and living in rural areas, which are exactly the group of the population at the highest risk of dementia. Urgent actions need to be taken to increase public awareness of dementia in China, especially among the vulnerable groups.

Our study confirmed that people with chronic conditions, especially those with diabetes, stroke, COPD, and arthritis, tended to have a higher prevalence of dementia, posing the importance of the prevention and treatment of these chronic diseases. Due to economic difficulties and low awareness of the disease among patients with dementia and their families, ~70–80% of them have not received treatment in China (34). The major challenge associated with the treatment of patients with dementia in China is the lack of a well-functioned dementia care system, which is further affected by the high cost of care and low levels of public awareness, and poor education among caregivers (35).

We found that daily green tea drinking, regular physical activity, reading books or newspapers, daily use of the internet, and frequent social activities were the protective factors for dementia, which are also reported in studies in China and other countries (36, 37). Our findings supported the importance of an active, intellectual, and socially integrated lifestyle among the elderly. In the most recent Healthy China 2030 action plan (38), promoting geriatric health is an important component, and specific actions included encouragement and support of the University for the Elderly, Activity Center for the elderly and Elderly Associations to organize healthy activities across the country.

One important strength of our study is the large sample size and stringent quality control. The study was carried out in 12 counties in 6 provinces across China. A total of 150 interviewers were involved, all of whom attended intensive training sessions and passed exams. A regional expert committee comprised of five provincial-level neurologists confirmed the clinical diagnosis of all cases in each province, and a central expert panel comprised of eight neurologists from the top hospitals in China worked together on difficult cases until a consensus was reached. With strong support from the central and local government of project sites, all the local project activities were well-organized, such as assigning the experienced interviewers to work early morning or night to coordinate with the schedule of participants, offering transportations to the study participants, and the provision of a green channel for all the clinical examinations by local hospitals. All these efforts contributed to the good data quality in this study. The structure of the weighted population was similar to the China Census 2010, proving the representativeness of our study population.

This study has some important limitations. First, temporal associations cannot be inferred due to the cross-sectional nature of the analysis, and recall and response biases might affect the prevalence estimates. Second, despite the weighting use and a high response rate, a limited number of provinces (only six) were selected to represent the national estimates. Older adults from remote rural areas and ethnic minority groups may have been left out. Third, our study participants were selected from the general community residents, with the exclusion of hospital and institutional communities, therefore the prevalence might be underestimated due to the institutionalization of some of the elderly individuals. Fourth, we were not able to identify the other subtypes of dementia, such as vascular disease, and cannot depict the whole picture of dementia prevalence. Finally, although we included many potentially associated factors in the logistic regression models, some of the variable categories were still crude. For example, we only asked the frequency of alcohol drinking, and information on the type and units of alcohol was not recorded.

Our study represents the most up-to-date study with large sample size and standard diagnosis in China to estimate the prevalence of dementia and AD in the general Chinese population. Based on the China National Statistics Bureau, there were 212.42 million Chinese population aged 60 years and over in 2015. If our prevalence estimates are correct, the number of people with dementia would be 8.96 million in China, posing a heavy burden to the family, society, and economy. This finding has serious implications for geriatric health and health services in the rapidly aging society in China. Our study lends further support to increase the awareness of dementia in the general public and to implement effective prevention and control measures on dementia in the Chinese population.

## Data Availability Statement

The raw data supporting the conclusions of this article will be made available by the authors, without undue reservation.

## Ethics Statement

The studies involving human participants were reviewed and approved by National Center for Chronic and Noncommunicable Disease Control and Prevention of Chinese Center for Disease Control and Prevention. The patients/participants provided their written informed consent to participate in this study.

## Author Contributions

ZW and DP contributed to conception and design of the study. QZ, YX, YD, ZD, YS, and JM contributed to the project execution. HZ organized the database. SQ performed the statistical analysis and wrote sections of the manuscript. PY wrote the first draft of the manuscript. All authors contributed to the article and approved the submitted version.

## Funding

This work was supported by the Major Programs of Public Health of China Ministry of Finance (131091106000150003).

## Conflict of Interest

The authors declare that the research was conducted in the absence of any commercial or financial relationships that could be construed as a potential conflict of interest.

## Publisher's Note

All claims expressed in this article are solely those of the authors and do not necessarily represent those of their affiliated organizations, or those of the publisher, the editors and the reviewers. Any product that may be evaluated in this article, or claim that may be made by its manufacturer, is not guaranteed or endorsed by the publisher.
