# Supplementary material for: Prevalence of Dementia in China in 2015: A Nationwide Community-Based Study
Source: Front Public Health. 2021 Nov 2;9:733314. doi: 10.3389/fpubh.2021.733314 (PMC8592944; doi:10.3389/fpubh.2021.733314)
Supplement: Supplementary file 1 [file Data_Sheet_1.docx]

**Appendix 1**

**Table 1: Population size in the study provinces in China, 2015**

|  | Population Number  (million) | Percentage in the total population  (%) | Population aged 60 years and above  (million) | Percentage in the total population aged 60 years and above (%) |
| --- | --- | --- | --- | --- |
| Beijing | 21.7 | 1.6 | 3.4 | 1.6 |
| Shanghai | 24.2 | 1.8 | 4.4 | 2.1 |
| Hubei | 58.5 | 4.3 | 9.5 | 4.5 |
| Sichuan | 82.0 | 6.0 | 16.7 | 7.9 |
| Guangxi | 48.0 | 3.5 | 7.1 | 3.3 |
| Yunnan | 47.4 | 3.5 | 6.0 | 2.8 |
| Total | 281.8 | 20.7 | 47.1 | 22.2 |

Total population in China in 2015 is 1373 million, 212 million were 60 years and over.

**Appendix 2: Weighting process**

We applied sampling weighting, non-response weighting and post-stratification weighting in the weighting process.

1. **Sampling weighting**

According to the sampling design of the survey, the sampling weigh for each individual is:

where Ws_1_ is the sampling weight for the selected county/district, W_s2_ is the sampling weight for the selected township/subdistrict, W_s3_ is the sampling weight for the selected village/neighborhood community, W_s4_ is the sampling weight for the selected participant. The equation for the sampling weight calculation at different levels is as follows:

1. county/district weighting

$$Ws1 =\frac{total number of counties/districts in the province}{number of selected counties/districts}$$

2. township/subdistrict weighting

$$Ws2 =\frac{number of people aged 60 years and above in all township/subdistrict in selected county/district}{number of people aged 60 years and above in selected township/subdistrict}$$

3. village/neighborhood community weighting

$$Ws3 =\frac{number of people aged 60 years and above in all village/neighborhood in selected township/subdistrict}{number of people aged 60 years and above in selected village/neighborhood community}$$

4. individual weighting

$$Ws4=\frac{number of people aged 60 years and above in selected village/neighborhood community}{number of selected people in the village/neighborhood community}$$

5. **Non-response weighting**

Non-response weighting Wr was calculated in the unit of village/neighborhood community and the equation is:

$$\mathrm{Wr}=\frac{number of selected people in the village/neighborhood community}{number of responded study participants in the village/neighborhood community}$$

1. **Post-stratification weighting**

The strata we considered for post-stratification weighting included: sex in two strata (men, women), ages in 7 strata (60-64, 65-69, 70-74, 75-79, 80-84, 85-89, 90+), region in two strata (urban, rural). The equation for strata weighting is:

$$W\alpha=\frac{number of people in strata k in whole population}{sum of sampling weighting in strata k in the sample}$$

And the final weighting is

After the weighting process, we compared the population distribution of our study participant with the China Census 2010 and the population pyramid showed similar age distribution.

Before weighting After weighting The China Census 2010

Figure 1 Comparison of the population of our study with China Census 2010

Table 2 Comparison of study population with China Census 2010

| Age group | Census  (%) | Unweighted survey population (%) | Weighted survey population (%) |
| --- | --- | --- | --- |
| 60-64 | 33.03 | 22.17 | 32.96 |
| 65-69 | 23.15 | 29.16 | 23.16 |
| 70-74 | 18.57 | 21.05 | 18.6 |
| 75-79 | 13.43 | 15.09 | 13.45 |
| 80-84 | 7.53 | 8.88 | 7.54 |
| ≧85 | 4.29 | 3.65 | 4.28 |

**Appendix 3**

**Table 3 Estimated prevalence of dementia and AD in Chinese population aged 65 and above**

|  | Dementia | |  | AD | |
| --- | --- | --- | --- | --- | --- |
|  | No. | Weighted prevalence  % (95%CI) |  | No. | Weighted prevalence  % (95%CI) |
| Overall | 667 | 5.34(3.08-7.61) |  | 406 | 2.94(2.10-3.78) |
| Sex |  |  |  |  |  |
| Men | 199 | 2.62(1.90-3.33) |  | 133 | 1.85(1.30-2.41) |
| Women | 468 | 7.87(3.78-11.97) |  | 273 | 3.94(2.65-5.24) |
| Location |  |  |  |  |  |
| Urban | 328 | 3.84(2.27-5.42) |  | 193 | 2.27(1.26-3.28) |
| Rural | 339 | 6.51(3.81-9.20) |  | 213 | 3.45(2.66-4.25) |
| Marital Status |  |  |  |  |  |
| Non-widowed | 422 | 4.34(2.50-6.17) |  | 252 | 2.27(1.62-2.92) |
| Widowed | 245 | 7.73(4.55-10.91) |  | 154 | 4.52(2.90-6.150) |
| Education, years |  |  |  |  |  |
| <1 | 412 | 8.76(4.06-13.47) |  | 241 | 4.64(2.92-6.37) |
| 1-6 | 141 | 2.85(1.85-3.86) |  | 91 | 1.53(0.93-2.14) |
| 7-9 | 67 | 2.39(0.95-3.83) |  | 40 | 1.44(0.39-2.49) |
| ≧9 | 47 | 2.96(2.49-3.43) |  | 34 | 2.38(1.90-2.86) |

**Appendix 4**

**Table 4 Estimated prevalence of dementia and AD in six provinces in China**

|  | No. of participants |  | Dementia | |  | AD | |
| --- | --- | --- | --- | --- | --- | --- | --- |
|  |  |  | No. | Weighted prevalence  % (95%CI) |  | No. | Weighted prevalence  % (95%CI) |
| Beijing | 4057 |  | 112 | 3.82(3.23-4.41) |  | 77 | 2.29(1.83-2.75) |
| Shanghai | 4020 |  | 74 | 1.55(1.17-1.93) |  | 53 | 1.07(0.75-1.39) |
| Hubei | 4034 |  | 127 | 3.57(3.00-4.15) |  | 75 | 2.40(1.93-2.87) |
| Guangxi | 3905 |  | 53 | 1.05(0.73-1.37) |  | 31 | 0.57(0.33-0.80) |
| Sichuan | 4065 |  | 221 | 6.25(5.50-7.00) |  | 120 | 3.04(2.51-3.56) |
| Yunnan | 4036 |  | 153 | 3.88(3.29-4.48) |  | 96 | 2.35(1.89-2.82) |

**Appendix 5**

Figure 2 Estimated prevalence of dementia stratified by previous diseases
